# Re-emergence of T lymphocyte-mediated synaptopathy in progressive multiple sclerosis

**DOI:** 10.3389/fimmu.2024.1416133

**Published:** 2024-06-07

**Authors:** Krizia Sanna, Antonio Bruno, Sara Balletta, Silvia Caioli, Monica Nencini, Diego Fresegna, Livia Guadalupi, Ettore Dolcetti, Federica Azzolini, Fabio Buttari, Roberta Fantozzi, Angela Borrelli, Mario Stampanoni Bassi, Luana Gilio, Gianluca Lauritano, Valentina Vanni, Francesca De Vito, Alice Tartacca, Fabrizio Mariani, Valentina Rovella, Alessandra Musella, Diego Centonze, Georgia Mandolesi

**Affiliations:** ^1^ Department of Systems Medicine, University of Rome Tor Vergata, Rome, Italy; ^2^ Ph.D. Program in Neuroscience, Department of Systems Medicine, Tor Vergata University, Rome, Italy; ^3^ Unit of Neurology, Istituto di Ricovero e Cura a Carattere Scientifico (IRCCS) Neuromed, Pozzilli, IS, Italy; ^4^ Synaptic Immunopathology Lab, Istituto di Ricovero e Cura a Carattere Scientifico (IRCCS) San Raffaele Roma, Rome, Italy; ^5^ Department of Human Sciences and Quality of Life Promotion, University of Rome San Raffaele, Rome, Italy

**Keywords:** secondary progressive multiple sclerosis, neurodegeneration, sphingosine receptor modulators, lymphocyte-mediated synaptopathy, neuroinflammation

## Abstract

**Background:**

Secondary progressive multiple sclerosis (SPMS) is defined by the irreversible accumulation of disability following a relapsing-remitting MS (RRMS) course. Despite treatments advances, a reliable tool able to capture the transition from RRMS to SPMS is lacking. A T cell chimeric MS model demonstrated that T cells derived from relapsing patients exacerbate excitatory transmission of central neurons, a synaptotoxic event absent during remitting stages. We hypothesized the re-emergence of T cell synaptotoxicity during SPMS and investigated the synaptoprotective effects of siponimod, a sphingosine 1-phosphate receptor (S1PR) modulator, known to reduce grey matter damage in SPMS patients.

**Methods:**

Data from healthy controls (HC), SPMS patients, and siponimod-treated SPMS patients were collected. Chimeric experiments were performed incubating human T cells on murine cortico-striatal slices, and recording spontaneous glutamatergic activity from striatal neurons. Homologous chimeric experiments were executed incubating EAE mice T cells with siponimod and specific S1PR agonists or antagonists to identify the receptor involved in siponimod-mediated synaptic recovery.

**Results:**

SPMS patient-derived T cells significantly increased the striatal excitatory synaptic transmission (n=40 synapses) compared to HC T cells (n=55 synapses), mimicking the glutamatergic alterations observed in active RRMS-T cells. Siponimod treatment rescued SPMS T cells synaptotoxicity (n=51 synapses). Homologous chimeric experiments highlighted S1P5R involvement in the siponimod’s protective effects.

**Conclusion:**

Transition from RRMS to SPMS involves the reappearance of T cell-mediated synaptotoxicity. Siponimod counteracts T cell-induced excitotoxicity, emphasizing the significance of inflammatory synaptopathy in progressive MS and its potential as a promising pharmacological target.

## 1 Introduction

Secondary progressive multiple sclerosis (SPMS) is a challenging clinical condition, defined by the gradual irreversible disability over at least 6–12 months in patients with a previous relapsing-remitting MS (RRMS) course (1). Diagnosed retrospectively, SPMS often evolves without MRI evidence of new white matter lesions, emphasizing the need for early recognition. Despite recent treatment options, a biomarker capturing the transition from remitting to progressive MS is lacking. Understanding immunological processes in this transition may aid early recognition and optimal treatment. Emerging concepts like relapse-associated worsening (RAW) and progression independent of relapse activity (PIRA) underscore differences in immunopathological mechanisms between relapsing and progressive MS. Clinical relapses, that occur in relapsing MS, are in fact essentially caused by white matter infiltration of circulating cells of the innate (e.g., macrophages) and adaptive (T and B lymphocytes) immune system, while SPMS pathophysiology is mainly driven by resident innate immune cells (microglia) and the compartmentalization of the adaptive immune system, essentially B cells, in meninges and perivascular spaces (1, 2).

We have recently demonstrated that during their relapsing phases, peripheral T lymphocytes from RRMS patients exacerbate excitatory transmission of central neurons through tumor necrosis factor (TNF) release (3). More specifically, CD3^+^ T lymphocytes isolated from the sera of RRMS patients increase the duration of spontaneous excitatory postsynaptic currents (sEPSCs) when placed on murine cortico-striatal brain slices during single neuron neurophysiological recordings, leading to secondary excitotoxic damage and striatal neuronal degeneration (heterologous chimeric ex-vivo MS model). The same TNF-mediated excitotoxic ability is retained by T lymphocytes sorted from splenocyte suspensions of EAE mice in the acute phase of the disease (homologous chimeric ex-vivo EAE model) (4), and cannot be observed when mouse brain slices are incubated with T cells taken from sera of healthy individuals, from MS patients in the remitting phase of the disease and from splenocytes of healthy mice (3, 4). These observations favor the hypothesis that, during MS disease activation, circulating T cells of RRMS patients can cause neuronal damage not only through white matter infiltration and axonal demyelination, but also through a largely independent and direct synaptototoxic mechanism in the grey matter.

Recognizing the relevance of inflammatory excitotoxic synaptopathy in progressive MS (5), we tested the challenging hypothesis that re-emergence of T lymphocyte-mediated synaptopathy, even after prolonged remission, could contribute to RRMS to SPMS transition. To further confirm the relevance of T lymphocyte-mediated synaptopathy in PIRA typical of SPMS, we explored the synaptoprotective effects of siponimod, a S1P receptor modulator demonstrated to effectively reduce accumulation of disability and grey matter damage in SPMS patients (6).

## 2 Methods

### 2.1 MS patients and healthy controls recruitment

A group of 17 consecutive SPMS patients was recruited at IRCCS Istituto Neurologico Mediterraneo (INM) Neuromed, Pozzilli (Isernia, Italy) during their clinical follow-up, and 6 healthy controls (HC). Key eligibility criteria were: age 18–65 years, a diagnosis of SPMS, documented moderate to advanced disability indicated with expanded disability status score (EDSS) score of 4–7 at screening, a history of RRMS, documented EDSS progression in the 2 years before the study, and no evidence of relapse and/or MRI activity in the 12 months before blood sampling. Key exclusion criteria included substantial immunological, cardiac, or pulmonary conditions, and uncontrolled diabetes.

Among the 17 SPMS patients, 7 received siponimod treatment, while 10 had discontinued disease-modifying therapies (DMT) for at least 1 year due to SPMS transition. Clinical characteristics recorded at blood sampling were age, sex, disease duration, and EDSS. Disease duration was defined as the interval relapsing between the first clinical episode compatible with MS and the time of blood sampling. Blood sampling of siponimod-treated patients was performed after 6 months of therapy.

### 2.2 Human T cells isolation

After blood withdrawal, peripheral blood mononuclear cells (PBMCs) were isolated by Ficoll histopaque gradient centrifugation, according to standard techniques, and soon frozen at -80°C (3). T cells were purified by magnetic immunosorting with FITC-CD3 antibody (RRID: AB_2725956, Miltenyi Biotec GmbH, Bergisch Gladbach, Germany) and microbeads-conjugated anti-FITC antibody (RRID: AB_244371, Miltenyi Biotec GmbH, Bergisch Gladbach, Germany) from defrosting PBMCs. Next, freshly isolated T cells (5 x 10^3^) were both diluted in artificial CSF (ACSF) and put in culture (RPMI medium, 1% penicillin/streptomycin, 1% glutamine, 10% fetal bovine serum and 5% of autologous human serum) for 24 h to perform chimeric experiments.

### 2.3 Mice and EAE induction

Chronic-progressive EAE was induced as previously described (7). Briefly, six-eight weeks old C57BL/6 (Charles River, Milan, Italy) female mice were active immunized with an emulsion containing 200 μg/animal of myelin oligodendrocyte glycoprotein peptide 35–55 (MOG35–55, 85% purity; Espikem, Prato, Italy) in Complete Freund’s Adjuvant (CFA; Difco, Los Altos, CA, USA) containing 8mg/ml lyophilized Mycobacterium tuberculosis (BD, M. Tubercolosis Des. H37 Ra, Sparks, USA) followed by intravenous administration of pertussis toxin (500 ng; Merck, Milan, Italy) on the day of immunization and two days post immunization (dpi). After EAE induction, animal clinical score (0, healthy; 1, limp tail; 2, ataxia and/or paresis of hindlimbs; 3, paralysis of hindlimbs and/or paresis of forelimbs; 4, tetraparalysis; 5, moribund or death) was recorded daily.

### 2.4 Murine T cell isolation

EAE mice were sacrificed at 15–20 dpi through cervical dislocation and the spleens were quickly removed and stored in sterile phosphate-buffered saline (PBS). After mechanical dissociation of the tissue, the cell suspension was passed through a 40-μm cell strainer (BD Biosciences, San Jose, CA, USA) to remove cell debris and centrifuged. The cell suspension obtained was subjected to magnetic cell sorting separation (CD3 microbeads kit; Miltenyi Biotec) to obtain a pure lymphocyte population. T cells were incubated overnight with siponimod (400 nM or 1μM) or vehicle; with selective S1P1R and S1P5R agonists (AUY954, 300 nM; A971432, 200 nM) or with vehicle, and also with siponimod in the presence of specific S1P1R antagonist (NIBR0213, 1 μM). About 5×10^3^ pure T cells were incubated onto striatal slices (derived from six-eight weeks old C57BL/6 female) for 60 min, in a total volume of 1 ml of oxygenated ACSF before the electrophysiological recordings (4, 8).

### 2.5 Electrophysiology

C57BL/6 (Charles River, Milan, Italy) female mice were sacrificed by cervical dislocation and the brains were removed. Then, cortico-striatal coronal slices (200 μm) were cut using a Vibratome (Leica VT1200 - Leica biosystems, Wetzlar, Germany) (5). A single slice was incubated for 1 hour with T cells and transferred (without inverting the side of incubation) to a recording chamber and submerged in a continuously flowing ACSF (32°C, 2–3 ml/min) gassed with 95% O2–5% CO2. The composition of the control ACSF was (in mM): 126 NaCl, 2.5 KCl, 1.2 MgCl_2_, 1.2 NaH_2_PO_4_, 2.4 CaCl_2_, 11 glucose, 25 NaHCO_3_. Only data from putative medium spiny projection neurons (MSNs) were included in this study. MSNs were identified for their morphological and electrophysiological properties (9, 10). Whole-cell patch-clamp recordings were made with borosilicate glass pipettes (1.8 mm o.d.; 2–3 MΩ), in voltage-clamp mode, at the holding potential of -80 mV. To study glutamate-mediated spontaneous excitatory postsynaptic currents (sEPSCs), the recording pipettes were filled with internal solution of the following composition (mM): K^+^-gluconate (125), NaCl (10), CaCl_2_ (1.0), MgCl_2_ (2.0), 1,2-bis (2-aminophenoxy) ethane-N,N,N,N-tetraacetic acid (BAPTA; 0.5), HEPES (19), GTP (0.3), Mg-ATP (1.0), adjusted to pH 7.3 with KOH. Picrotoxin (50 µM) was added to the perfusing solution to block GABA_A_-mediated transmission. The detection threshold of sEPSCs was set at twice the baseline noise. Offline analysis was performed on spontaneous synaptic events recorded during fixed time epochs (1–2 min, three to five samplings), sampled every 5 or 10 min. Only cells that exhibited stable frequencies in control (less than 20% changes during the control samplings) were used for analysis. For kinetic analysis, events with peak amplitude between 5 and 40 pA were grouped, aligned by half-rise time, normalized by peak amplitude and averaged to obtain rise times and decay times.

Synaptic events were stored using PCLAMP (Axon Instruments - Molecular Devices, San Jose, CA, USA) and analysed offline on a personal computer with Mini Analysis 6.0.7 (Synaptosoft, Leonia, NJ, USA) software. For each parameter analyzed, one to six cells per animal were recorded. Two to five animals per group were used.

### 2.6 MS T-cell heterologous chimeric experiments

Purified T cells (5 x 10^3^) were incubated on the surface of a single murine corticostriatal slice for 60 minutes. To minimize the potential impact of biological variations in glutamatergic transmission, chimeric experiments were conducted by incubating T cells from the same patient on slices obtained from at least two animals (3).

### 2.7 EAE T-cell homologous chimeric experiments

Purified T cells (5 x 10^3^) were incubated on the surface of a single murine corticostriatal slice for 60 minutes, as previously described (4, 8). To minimize the potential impact of biological variations in glutamatergic transmission, chimeric experiments were conducted by incubating T cells of the same EAE mice on slices from at least two healthy animals. In a subgroup, corticostriatal slices were co-incubated with EAE T cells and S1P modulators. Based on EC50 values (8, 11–14) we selected the following concentration: Siponimod (1 μM), AUY954 (300 nM); A971432 (200 nM); NIBR0213 (1 μM). All the drugs were dissolved in DMSO (0.1–0.25% final concentration).

### 2.8 Ethics

The clinical retrospective study was carried out in compliance with the Declaration of Helsinki principles and was approved by the Institutional Review Board (CE Oct. 26th, 2017; NCT03217396 recorded in https://clinicaltrials.gov/) of the IRCCS Istituto Neurologico Mediterraneo (INM) Neuromed, Pozzilli (Isernia, Italy). All patients with SPMS gave their written informed consent to participate in the study.

Animal experiments described in this study were conducted according to the guidelines set by the Internal Institutional Review Committee, the European Directive 2010/63/EU and the European Recommendations 526/2007 and the Italian D. Lgs 26/2014. All the efforts were made to minimize the number of animals used and their suffering. In particular, when animals experienced hindlimb weakness, moistened food and water were made easily accessible to the animals on the cage floor. Mice with hindlimb paresis received food by gavage during the entire procedure. In the rare presence of a tetraparalyzed animal, death was provided.

### 2.9 Statistical analysis

Sample size calculation for heterologous T cell chimeric *ex-vivo* model: by *a priori* power analysis (G*Power 3.1.9.2 software) of the difference between two independent means (two-tail t-test; α = 0.05; 1-β = 0.8) and on the basis of previous and preliminary data (3), we estimated n=42 cells for electrophysiological experiments (effect size d=0.62, mean difference 0.5; SD deviation=0.8). To account for biological differences in glutamatergic transmission recordings from different animals, chimeric experiments were performed by incubating T cells of the same patient on brain slices from at least two animals. Considering three cells/animals for sEPSC recording and two mice for each patient, we estimated N=7 human samples for each experimental group and N=14 mice. Similarly, for homologous T cell chimeric *ex-vivo* EAE model (4), we estimated n=10 cells for sEPSC recording (effect size d=1.35; mean difference 0.5; standard deviation=0.4). Considering three cells/animal electrophysiological experiments and two mice for each EAE, we estimated N=4 EAE mice and N=8 C57B/L6 mice for each experimental group. Statistical analysis was performed using Prism GraphPad 9.0. Differences between two groups were analyzed using two-tailed Unpaired Student’s t test. The significance level was established at P<0.05. Multiple comparisons were performed by ANOVA followed by Tukey’s *post hoc* test. Data were presented as the mean ± S.E.M, unless otherwise specified. The exact statistical test used for each experiment and its details can be found in the figure legends and in **Supplementary Table 1**.

## 3 Results

### 3.1 Non-active SPMS T lymphocytes alter sEPSC kinetics in the heterologous chimeric *ex vivo* model

**Figure 1:**
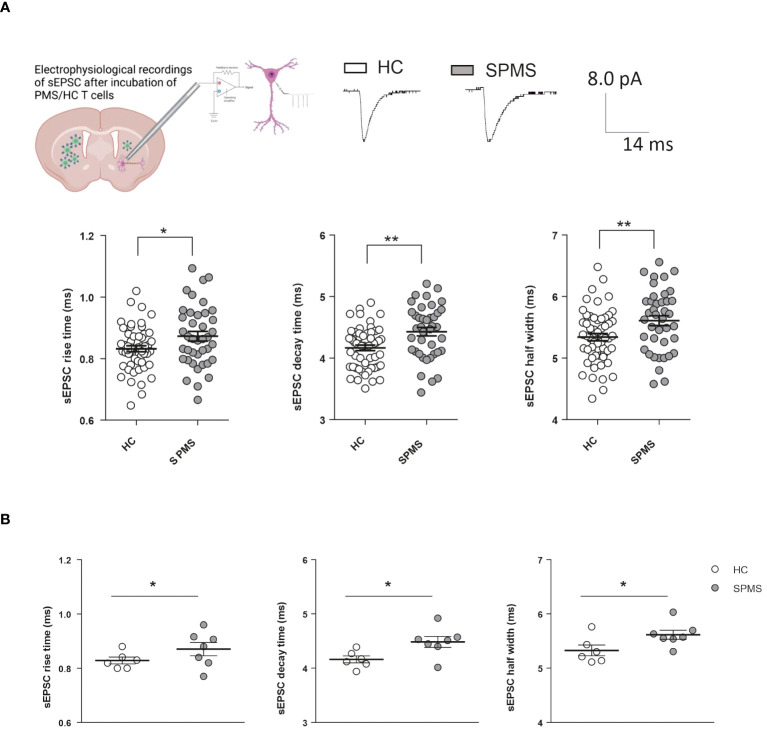
T lymphocytes from SPMS patients induce excitotoxic effects in a heterologous chimeric *ex vivo* model. **(A)** Schematic representation of heterologous T-cell chimeric MS model. Voltage-clamp recordings from MSNs of murine corticostriatal slices incubated with HC-T cells or SPMS-T cells. Representative peaks of electrophysiological recordings in the two experimental conditions are shown in the top panel. The graphs show the kinetic properties of sEPSCs in the two experimental conditions. The rise time, decay time, and half-width were altered in SPMS patients compared to HS (HC: N =6, n =55 cells, SPMS: N =7, n =40 cells). Each dot in the graphs represents the value of a single cell time rise, decay time, and half-width. HC and SPMS populations are labeled by white and grey color dots, respectively. **(B)** The differences observed in graphs A were maintained even considering the mean values of the parameters for each subject. Dotted lines refer to control conditions. Data are expressed as mean ± SEM. Statistical analysis was performed by Unpaired T-test; * p < 0.05; ** p < 0.01; *** p < 0.001. N represents numbers of patients and n represents numbers of cells.

To investigate the role of adaptive immunity on synaptic transmission in SPMS, we performed electrophysiological recordings from medium spiny neurons (MSNs) of the striatum of healthy mice in the presence of T cells taken from non-active SPMS patients and HC. Clinical and demographic information of the recruited participants is described in **Table 1**. As observed in **Figure 1A**, all the kinetic parameters of sEPSC (rise time, decay time, and half-width) were significantly increased by SPMS lymphocytes compared to HC (HC: N =6, n =55 cells, SPMS: N =7, n =40 cells, rise time p=0.022, decay time p=0.001 and half-width p=0.007, Unpaired T-test), resembling the excitotoxic damage observed in the experimental models of MS (3, 4). These results were supported by the data analysis calculating the mean kinetic value for each patient. The difference between T cells derived from HC and SPMS was completely maintained for each kinetic parameter (**Figure 1B**, HC: N =6, n =55 cells, SPMS: N =7, n =40 cells, rise time p=0.023, decay time p=0.025 and half-width p=0.028, Unpaired T-test), confirming the influence of SPMS disease on the synaptic perturbation mediated by T lymphocytes.

**Table 1:** Clinical and demographic features of MS patients and healthy controls.

	SPMS patients (untreated) N=10	SPMS patients (treated with Siponimod) N=7	HC N=6	P value
Age, years (Median,IQR)	53 (41.5–63.5)	54.0 (45.0–55.0)	47.5 (46.8–49.5)	0.675
Sex (F/M) (%)	4/10 (40.0)	6/7 (85.7)	3/6 (50.0)	0.082
Disease duration, years (Median,IQR)	16.5 (11.8–19.5)	20.0 (16.0–26.0)	–	0.133
Progressive phase duration, years (Median,IQR)	6.3 (3.8–9.5)	4.0 (3.0–7.0)	–	0.270
EDSS, value (Median,IQR)	6.0 (5.5–6.5)	4.5 (4.0–6.5)	–	0.109
OCB (yes/N tot) (%)	9/10 (90)	7/7 (100)	–	0.588
MRI activity (yes/N tot) (%)	0/10 (0)	0/7 (100)	–	–

SPMS, secondary progressive multiple sclerosis; HC, healthy controls; IQR, interquartile range; F, female; M, male; EDSS, Expanded Disability Status Scale; OCB, oligoclonal bands; MRI, magnetic resonance imaging. Statistical analyses were assessed using Mann-Whitney for 2 independent samples, and Kruskall-Wallis for 3 independent samples.

Altogether, these results indicate that, even in the absence of clinical and radiological activity, SPMS T cells promote excitotoxic damage by enhancing glutamate transmission in central neurons.

### 3.2 Siponimod treatment of SPMS patients reverses T lymphocyte-induced sEPSC kinetic abnormalities

**Figure 2:**
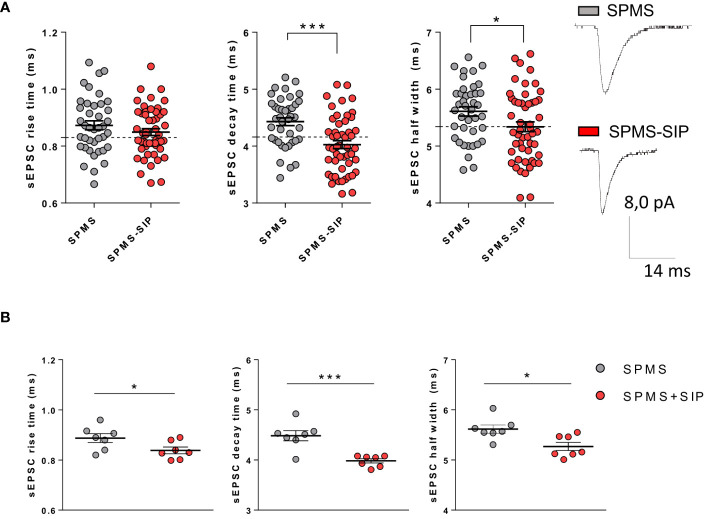
Siponimod is able to normalize SPMS T cell-induced synaptic abnormalities in a heterologous chimeric *ex vivo* model. Voltage-Clamp recordings were performed in the presence of T cells isolated from the peripheral blood of SPMS patients and SPMS patients treated with siponimod for at least six months. **(A)** Siponimod treatment recovered T cell-induced synaptotoxicity in SPMS patients (SPMS: N =7, n =40 cells; SPMS-SIP: N =7, n=51 cells). The electrophysiological traces are representative of the sEPSC mean peak of each experimental condition. Each dot represents the value of a single cell time rise, decay time, and half-width of the two experimental groups. SPMS and SPMS-SIP populations are labeled by grey and red color dots, respectively. **(B)** The differences observed in graphs A were maintained even considering the mean values of the parameters for each subject. Dotted lines refer to control conditions. Data are expressed as mean ± SEM. Statistical analysis was performed by Unpaired T-test; ** p < 0.05; ** p < 0.01; *** p < 0.001. N represents numbers of patients and n represents numbers of cells.

To address the potential beneficial effect of siponimod on T cell-induced synaptopathy in SPMS, we recorded glutamatergic transmission from murine striatal neurons in the presence of T cells taken from SPMS patients treated with siponimod for at least 6 months. Demographic information about the population examined in this study is reported in **Table 1**. The sEPSC decay time and half-width parameters recorded following incubation of brain slices with T lymphocytes from siponimod-treated SPMS patients were recovered compared to T cells taken from untreated SPMS individuals (**Figure 2A**, SPMS: N =7, n =40 cells; SPMS-SIP: N =7; n =51 cells; rise time p=0.230, decay time p<0.0001, half-width p=0.028, Unpaired T-test), showing values similar to the control condition (dotted lines in the figure). These results were confirmed by the data analysis calculating the mean values of the parameters for each participant (**Figure 2B**, SPMS: N =7; SPMS-SIP: N =7; rise time p=0.047, decay time p=0.0006, half-width p=0.011, Unpaired T-test) and show the beneficial effect of siponimod on T cell-induced synaptotoxicity.

### 3.3 Siponimod abolishes the synaptotoxic effects of EAE T lymphocytes in homologous chimeric *ex vivo* model

**Figure 3:**
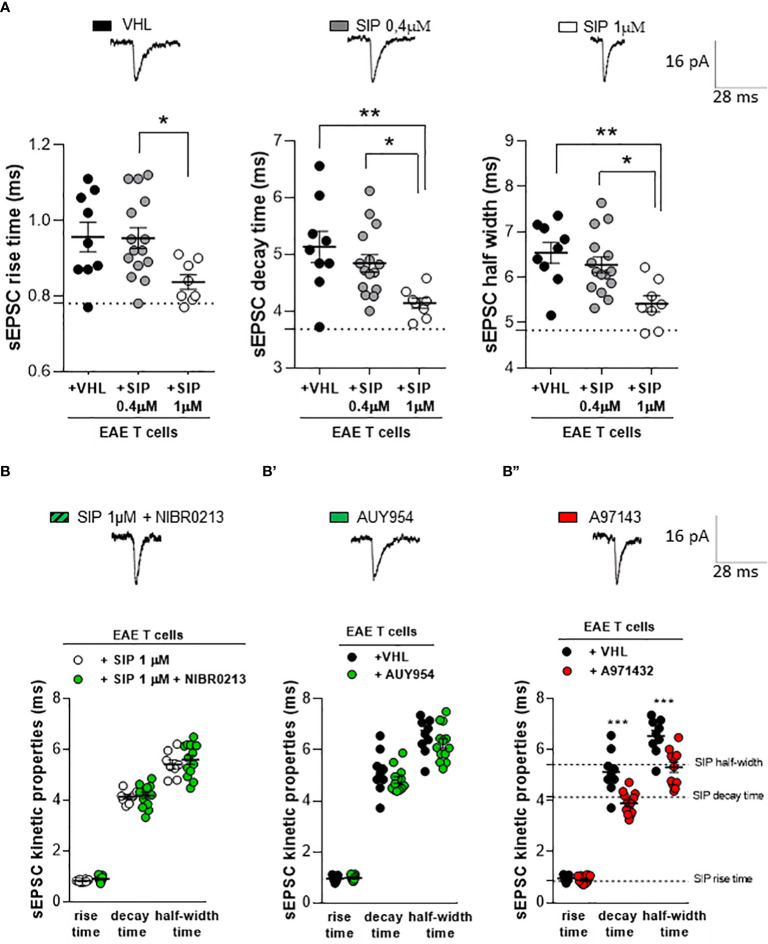
The beneficial effect of siponimod is mediated by a specific modulation of S1P5 receptor in homologous chimeric *ex vivo* model. **(A)** The enhancement of sEPSC decay time and half-width, typically induced by EAE lymphocytes, was significantly reduced by *in vitro* pre-treatment of EAE T cells with siponimod (1 μM). Conversely, 0,4 μM of siponimod pre-treatment did not induce any effect. Electrophysiological traces above the graphs, represent examples of sEPSC derived from the three experimental conditions. Dotted lines refer to control conditions (EAE-VHL: N =4, n =9 cells, EAE-SIP 0.4μM: N =4, n =15 cells; EAE-SIP 1 μM: N =4, n =8). **(B)** The co-incubation of the S1P1 receptor antagonist NIBR0213 (1 μM) and siponimod (1 μM) on EAE T cells did not affect the striatal sEPSC kinetic parameters compared to siponimod-treated EAE T cells. Dotted lines refer to vehicle condition (EAE-SIP 1μM: N=4, n=8 cells, EAE-SIP 1μM+NIBR0213: N=4, n=13 cells). **(B’)** The incubation of the S1P1 receptor agonist, AUY954 (300 nM) on EAE T cells was unable to recover the alterations of sEPSC kinetic parameters, typically induced by EAE T cells. Dotted lines refer to siponimod condition (EAE-VHL: N=4, n=9 cells, EAE-AUY954: N=4, n=13 cells). **(B’’)** The incubation of EAE T cells with S1P5 receptor agonist, A971432 (200 nM), recovered EAE T cell-dependent synaptic excitotoxicity, compared to vehicle-treated EAE T cells. Dotted lines refer to siponimod condition (EAE-VHL: N=4, n=9 cells, EAE-A971432: N=4, n=12 cells). The electrophysiological traces, above the graphs, are representative of the sEPSC mean peak of each experimental condition. Data are expressed as mean ± SEM. Statistical analysis was performed by One-way ANOVA when comparing three groups, and Unpaired T-test when comparing two groups; * p < 0.05; ** p < 0.01; *** p < 0.001. N represents numbers of EAE mice and n represents numbers of cells.

To address the receptor mechanism involved in the siponimod-mediated rescue of inflamed synapses, we investigated the T cell-induced synaptic alterations in the homologous ex vivo model (4). CD3^+^ T cells were isolated from the spleen of EAE mice (15–20 dpi) and cultured overnight with siponimod (two different concentrations: 0.4 μM and 1 μM) or vehicle. Then, EAE T cells were incubated (for 1h) on cortico-striatal slices of healthy mice to record sEPSCs. EAE vehicle T cells induced an increase of sEPSC kinetic, as previously demonstrated (4), and siponimod treatment rescued the T cell-induced synaptic alteration (**Figure 3A**). In particular, EAE T cells treated with 1 μM siponimod, but not 0.4 μM rescued the decay time and half-width of sEPSC, leading to full synaptic recovery (**Figure 3A**). Siponimod (1 μM) treatment exerted a protective effect, compared to siponimod 0.4 μM, showing reduced rise time, decay time, and half-width (EAE-VHL: N =4, n =9 cells, EAE-SIP 0.4 μM: N =4, n =15 cells; EAE-SIP 1 μM: N =4, n =8; rise time p=0.026, decay time p=0.007, half width time p=0.003, one-way ANOVA).

### 3.4 S1P5Rs are involved in the recovery of lymphocyte-mediated excitotoxicity by siponimod

To identify the sphingosine receptor subtypes involved in the synaptic rescue of glutamatergic toxicity induced by siponimod-treated T cells, we performed electrophysiological experiments with the use of agonists and antagonists of S1PRs. Since the S1P1R is the most abundant receptor expressed on T cells (15), we started by studying this receptor subtype. We first co-incubated EAE T cells overnight with a selective S1P1R antagonist, NIBR0213 (1 μM), and with siponimod (1 μM). Then, we incubated EAE T cells on cortico-striatal slices of healthy mice to perform electrophysiological recordings. As shown in **Figure 3B**, NIBR0213 failed to antagonize siponimod-mediated synaptic rescue (EAE-SIP 1μM: N=4, n=8 cells, EAE-SIP 1μM+NIBR0213: N=4, n=13 cells; rise time p=0.069, decay time p=0.741, half-width p=0.510, Unpaired T-test), suggesting that the receptor subtype S1P1 is not involved in the beneficial effect of siponimod. In agreement with these observations, EAE T cells treated with the selective S1P1R agonist, AUY954 (300 nM) and incubated on striatal slices of healthy mice failed to modulate synaptic properties (**Figure 3B’**, EAE-VHL: N=4, n=9 cells, EAE-AUY954: N=4, n=13 cells; rise time p=0.692, decay time p=0.270, half-width p=0.343, Unpaired T-test).

To assess the involvement of S1P5R, we investigated the effect of selective S1P5R agonist A971432 (200 nM). As shown in **Figure 3B’’**, the incubation with A971432 was able to rescue the striatal glutamatergic alterations mediated by EAE T cells. In particular, the sEPSC decay time and half-width were recovered, reaching comparable values to that of siponimod 1 μM (**Figure 3B’’**: EAE-VHL: N=4, n=9 cells, EAE-A971432: N=4, n=12 cells; rise time p=0.030, decay time p=0.0003, half-width p=0.001, Unpaired T-test).

The lack of a selective S1P5R antagonist prevented us from further exploring the involvement of this receptor subtype in the siponimod effect. These results indicate that siponimod is able to recover the EAE T cells synaptotoxicity through the specific involvement of S1P5 receptor subtype.

## 4 Discussion

MS is characterized by a chronic infiltration of innate and adaptive immune cells in the meninges and connective tissue spaces of the brain. This process is even more marked in the progressive phases of the disease (16). The compartmentalization of neuroinflammation in the CNS is crucial for the pathophysiology of SPMS (1), and it is considered the cause of the drug resistance that characterizes this disease phenotype (1). Despite the presence of T lymphocytes in CNS infiltrates, current hypotheses on SPMS pathophysiology mainly focus on neurodegenerative processes mediated by resident innate immune cells (17) and by B lymphocytes organized in pseudo-follicular structures (18).

The pathological interaction between inflammation and synaptic transmission identifies the paradigm of the so called inflammatory synaptopathy, which is considered a pathological feature of both MS and EAE (19–21). Glutamate-mediated excitotoxicity is one of the pathogenetic mechanisms underlying neurodegeneration in MS (22, 23) and, in fact, long-lasting increase of neuronal sensitivity to glutamate contributes to dendritic spine degeneration and neuronal loss in these conditions (3, 24).

To explore the effect of T lymphocytes from RRMS patients on glutamate mediated synaptic transmission, we recently developed a chimeric model of MS (3). This model revealed that human T lymphocytes, exclusively during the active phase of RRMS, significantly increased sEPSC kinetics of striatal neurons (3), mimicking the synaptic abnormalities typical of EAE. Despite the insensitivity of glutamate synapses to T lymphocytes during clinical and radiological remission of RRMS patients, here we demonstrated that T lymphocytes re-acquire synaptotoxic abilities after clinical transition to SPMS even in the absence of MRI evidence of inflammatory disease reactivation. These synaptic alterations could be mediated by a local inflammatory reaction induced by SPMS T cells, maybe involving TNF signaling. Clinical and preclinical studies have consistently implicated this proinflammatory cytokine in the alterations of excitatory synaptic transmission (3, 4, 25). Notably, elevated levels of TNF have been detected in the serum, CSF, and T cells of MS patients (3, 26, 27), and have been associated with excitotoxic synaptic alterations induced by CSF from PMS patients and by T cells from RRMS (3, 28). Further experiments should be performed to investigate the role of TNF-mediated excitotoxicity in PMS patients.

This study shed new light on the pathophysiology of SPMS and might be useful for the development of an early biomarker of this disease condition, whose diagnosis is always retrospective and delayed. Our data also contribute to rethinking the relevance of T lymphocytes in the pathophysiology of SPMS, supporting current pharmacological and clinical research (29).

As demonstrated in the phase 3 clinical trial EXPAND, siponimod slows disability progression in patients with SPMS (6, 30, 31). Despite S1PR modulators primarily act in MS by reducing lymphocyte trafficking into the CNS (6, 29), previous clinical and preclinical evidence demonstrated that siponimod, the first DMT approved for the treatment of SPMS, can exert neuroprotective effects and influence synaptic transmission even independently of its action on peripheral immune cells. Direct intracerebroventricular delivery of siponimod, in fact, ameliorated the clinical deficits and the associated synaptopathy of EAE mice (32). In particular, this treatment selectively rescued GABAergic transmission alterations typical of the EAE striatum, likely due to reduced local inflammatory reaction and increased survival of parvalbumin-positive GABAergic interneurons, which are highly sensitive to EAE and MS processes (32, 33).We also demonstrated that ozanimod, a S1PR 1,5 modulator approved for the treatment of RRMS, was able to dampen striatal glutamatergic alterations in EAE mice as well as in EAE T lymphocytes homologous chimeric ex-vivo model (8).

In the present study, we demonstrated that the beneficial effect of siponimod extends to the synaptotoxic effects of T lymphocytes of SPMS patients, providing a possible further explanation of the neuroprotective role of this compound in SPMS. In particular, we showed that the sensitivity of glutamate synapses to T cells from SPMS patients was rescued in siponimod-treated SPMS patients and, in parallel, that siponimod treatment of EAE T lymphocytes recovered their synaptotoxic properties. Furthermore, we also characterized the S1PR subtype involved in the effects of siponimod on T lymphocyte-mediated neurotoxicity. We found, in fact, that pharmacological activation of S1P5Rs was able to mimic the effects of siponimod, while S1P1Rs were not involved in this action.

**Figure 4:**
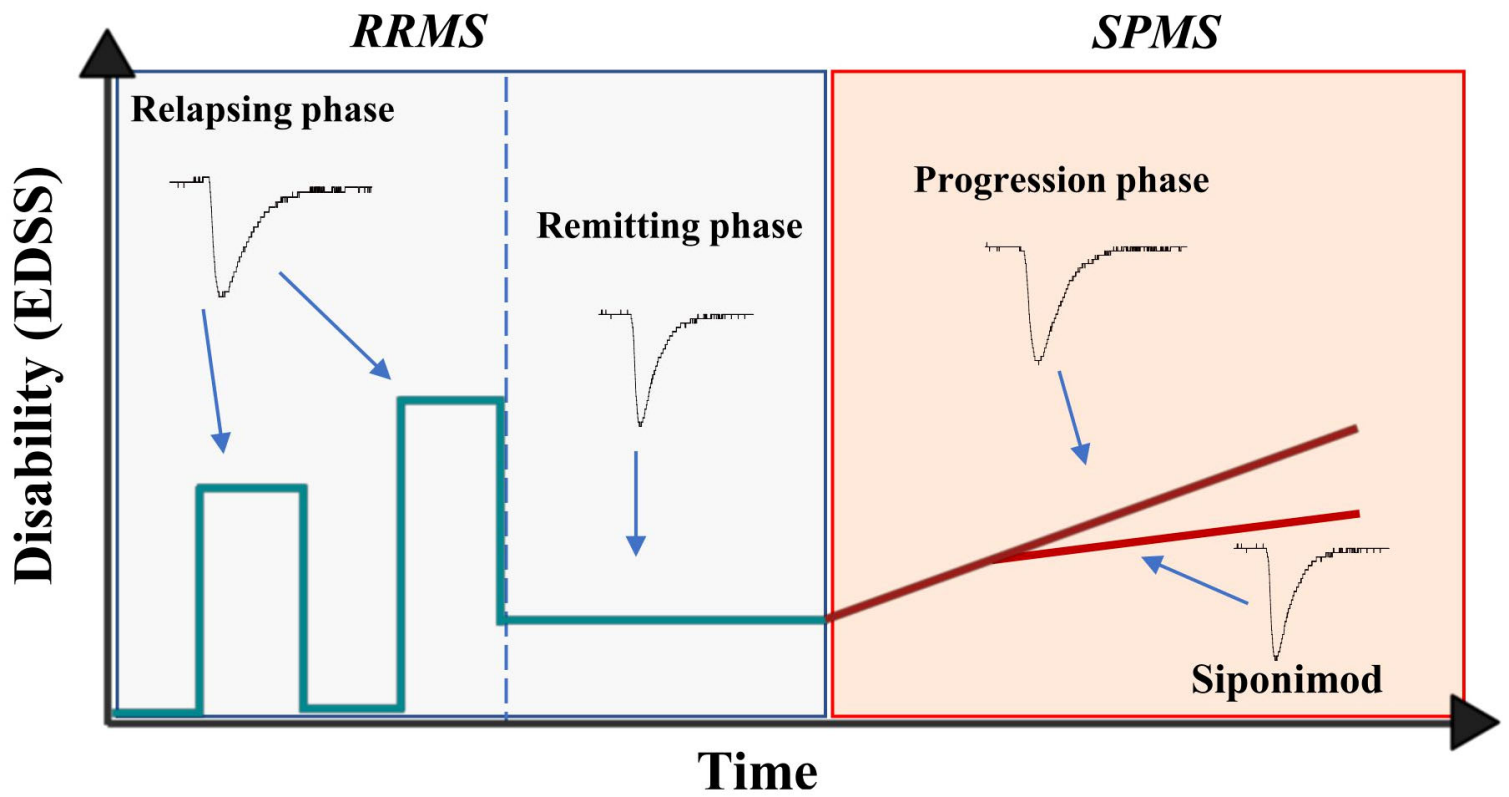
Representative image showing the progression of multiple sclerosis disability and T-cell mediated synaptopathy during the course of the disease. During the relapsing phase of RRMS, the disability (EDSS) of patients increase and it is accompanied by exacerbation of T lymphocyte-induced excitatory transmission, a synaptotoxic ability absent during the remitting stages of the disease. The synaptotoxic ability of T lymphocytes re-emerges with disease progression, independently of disease activity, and siponimod is able to rescue this synaptic alteration.

This study shows for the first time the ability of T lymphocytes of SPMS patients to alter excitatory transmission at central synapses, suggesting that PIRA, that characterizes this form of the disease, involves chronic neuronal excitotoxic damage mediated by adaptive immune cells. T lymphocyte-induced synaptopathy, in fact, also characterizes the relapsing phases of RRMS (3), normally limited to a few weeks or months, and is instead absent during the longer lasting (months or years) remission stages. Transition to SPMS is accompanied by the reappearance of this synaptic effect by T lymphocytes, likely contributing to neurodegeneration processes due to its chronic maintenance (**Figure 4**).

However, one limitation of the current study is the unequal distribution of SP-MS patients between the two treatment groups, with a higher proportion of females in the siponimod-treated SP-MS group compared to untreated patients. To enhance the statistical robustness of our findings, it would be essential to consider expanding the population of siponimod-treated individuals as a prospective avenue. Furthermore, in the present paper we characterized the role of T CD3+ cells in terms of synaptopathy during SPMS. It is acknowledged that during the progressive phases of the disease, the prevalence of CD8+ cells in both CNS lesions and peripheral compartments may significantly contribute to neuroinflammatory degeneration in SP-MS brains. Therefore, further investigation into the functional dynamics of T cell subpopulations is deemed necessary to elucidate the intricate interplay between the T- and B-cell compartments in MS and EAE.

The evidence that siponimod, that effectively contrasts SPMS evolution, also contrasts T lymphocyte-induced excitotoxicity confirms the relevance of inflammatory synaptopathy in progressive MS and helps to clarify the mechanisms by which this DMT exerts its neuroprotective effect.

## Data availability statement

The original contributions presented in the study are included in the article/**Supplementary Material**. Further inquiries can be directed to the corresponding author.

## Ethics statement

The studies involving humans were approved by Institutional Review Board (CE Oct. 26th, 2017; NCT03217396 recorded in https://clinicaltrials.gov/) of the IRCCS Istituto Neurologico Mediterraneo (INM) Neuromed, Pozzilli (Isernia, Italy). The studies were conducted in accordance with the local legislation and institutional requirements. Written informed consent for participation in this study was provided by the participants’ legal guardians/next of kin. The animal study was approved by Internal Institutional Review Committee, the European Directive 2010/63/EU and the European Recommendations 526/2007 and the Italian D. Lgs 26/2014. The study was conducted in accordance with the local legislation and institutional requirements.

## Author contributions

KS: Writing – original draft, Methodology, Formal analysis. ABr: Writing – original draft, Data curation. SB: Writing – review & editing, Methodology, Formal analysis. SC: Writing – review & editing, Methodology, Formal analysis. MN: Writing – review & editing, Methodology, Formal analysis. DF: Writing – review & editing, Methodology, Formal analysis. LGu: Writing – review & editing, Methodology, Formal analysis. ED: Writing – review & editing, Data curation. FA: Writing – review & editing, Data curation. FB: Writing – review & editing, Data curation. RF: Writing – review & editing, Data curation. ABo: Writing – review & editing, Data curation. MS: Writing – review & editing, Data curation. LGi: Writing – review & editing, Data curation. GL: Writing – review & editing, Data curation. VV: Writing – review & editing, Methodology, Formal analysis. FV: Writing – review & editing, Methodology, Formal analysis, Data curation. AT: Writing – review & editing, Formal analysis. FM: Writing – review & editing, Formal analysis. VR: Writing – review & editing, Data curation. AM: Writing – review & editing, Supervision, Data curation. GM: Writing – original draft, Validation, Supervision, Investigation, Data curation. DC: Writing – original draft, Validation, Supervision, Project administration, Investigation, Funding acquisition.
