# Supplementary material for: Re-emergence of T lymphocyte-mediated synaptopathy in progressive multiple sclerosis
Source: Front Immunol. 2024 Jun 7;15:1416133. doi: 10.3389/fimmu.2024.1416133 (PMC11190089; doi:10.3389/fimmu.2024.1416133)
Supplement: Supplementary file 1 [file Table_1.pdf]

Supplementary Table 1: Statistical Analysis

Figure	Pre hoc	Post hoc	N / n (number of animals / samples)
**1 A**	Unpaired two-tailed Student's t test:<br>1) rise time: t=2.325, p=0.0223<br>2) decay time: t=3.351, p=0.0012<br>3) half width: t= 2.748, p=0.0072<br>Df: 93		N: HC 6; SPMS 7<br>n (number of cells): HC 55; SPMS 40
**1 B**	Unpaired two-tailed Student's t test:<br>1) rise time: t=2.643, p=0.0229<br>2) decay time: t=2.594, p=0.0249<br>3) half width: t= 2.523, p=0.0283<br>Df: 11		N: HC 6; SPMS 7<br>n (number of cells): HC 55; SPMS 40
**2 A**	Unpaired two-tailed Student's t test:<br>1) rise time: t=1.208, p=0.2303<br>2) decay time: t=4.088, p<0.0001<br>3) half width: t= 2.238, p=0.0277<br>Df: 89		N: SPMS 7; SPMS-SIP 7<br>n (number of cells): SPMS 40; SPMS-SIP 51
**2 B**	Unpaired two-tailed Student's t test:<br>1) rise time: t=2.213, p=0.0470<br>2) decay time: t=4.584, p=0.0006<br>3) half width: t= 2.993, p=0.0112<br>Df: 12		N: SPMS 7; SPMS-SIP 7<br>n (number of cells): SPMS 40; SPMS-SIP 51
**3 A**	One-way ANOVA:<br>1) rise time: F(2,29)=4.163, p=0.0257<br>2) decay time: F(2,29)=5.896, p=0.0071<br>3) half width: F(2,29)=7.158, p=0.0030	Tukey multiple comparisons test	N: EAE-VHL 4; EAE-SIP 0.4 μM 4; EAE-SIP 1 μM 4<br>n (number of cells): EAE-VHL 9; EAE-SIP 0.4 μM 15; EAE-SIP 1 μM 8
**3B**	Unpaired two-tailed Student's t test:<br>1) rise time: t=1.929, p=0.0689<br>2) decay time: t=0.3352, p=0.7412<br>3) half width: t=0.6705, p=0.5106<br>Df: 19		N: EAE-SIP 1 μM 4; EAE-SIP 1 μM+NIBR0213 4<br>n (number of cells): EAE-SIP 1 μM 8; EAE-SIP 1 μM+NIBR0213 13
**3B’**	Unpaired two-tailed Student's t test:<br>1) rise time: t=0.4015, p=0.6923<br>2) decay time: t=1.135, p=0.2700<br>3) half width: t=0.9697, p=0.3438<br>Df: 20		N: EAE-VHL 4; EAE-AUY957 4<br>n (number of cells): EAE-VHL 9; EAE-AUY957 13
**3B’’**	Unpaired two-tailed Student's t test:<br>1) rise time: t=1.064, p=0.3007<br>2) decay time: t=4.390, p=0.0003<br>3) half width: t=4.032, p=0.0007<br>Df: 19		N: EAE-VHL 4; EAE-A971432 4<br>n (number of cells): EAE-VHL 9; EAE-A971432 12
